# COVID-19 lockdown policy and heterogeneous responses of urban mobility: Evidence from the Philippines

**DOI:** 10.1371/journal.pone.0270555

**Published:** 2022-06-30

**Authors:** Yi Jiang, Jade R. Laranjo, Milan Thomas

**Affiliations:** Economic Research and Regional Cooperation Department, Asian Development Bank, Manila, Philippines; Gebze Teknik Universitesi, TURKEY

## Abstract

Throughout 2020, national and subnational governments worldwide implemented nonpharmaceutical interventions (NPIs) to contain the spread of COVID-19. These included community quarantines, also known as lockdowns, of varying length, scope, and stringency that restricted mobility. To assess the effect of community quarantines on urban mobility in the Philippines, we analyze a new source of data: cellphone-based origin-destination flows made available by a major telecommunication company. First, we demonstrate that mobility dropped to 26% of the pre-lockdown level in the first month of lockdown and recovered and stabilized at 70% in August and September of 2020. Then we quantify the heterogeneous effects of lockdowns by city’s employment composition. A city with 10 percentage points more employment share in work-from-home friendly sectors is found to have experienced an additional 2.8% decrease in mobility under the most stringent lockdown policy. Similarly, an increase of 10 percentage points in employment share in large and medium-sized firms was associated with a1.9% decrease in mobility on top of the benchmark reduction. We compare our findings with cross-country evidence on lockdowns and mobility, discuss the economic implications for containment policies in the Philippines, and suggest additional research that can be based on this novel dataset.

## Introduction

### Context

To control the spread of COVID-19, national and subnational governments in nearly all countries have instituted non-pharmaceutical interventions (NPIs) over the past two years [1]. These public health measures have been imposed and enforced most strongly in high-density urban centers, and include closing schools and other public venues, instituting curfews, and limiting business activities and size of social gatherings. The primary goal of such policies is to reduce mobility of people and contain the pandemic by limiting human contact.

The effects of those lockdown policies on mobility in cities have been studied for several high-income countries. Those studies typically use big data sources to analyze how policies of differing stringency levels affect vehicular and non-vehicular mobility over time and by locational characteristics. This mobility-focused research agenda is highly relevant because many studies have confirmed that mobility is closely associated with public health outcomes such as infection, hospitality, and mortality [2,3]. On the other hand, mobility restrictions have been found to adversely affect economic activity [4–6].

However, there is less empirical evidence on how mobility has responded to lockdowns in lower-income countries, where lockdowns have often lasted longer, entailed higher enforcement costs, and caused greater damage to the economy. One reason for missing evidence in these countries is lack of frequent, high-quality data on people’s travel. However, big data collected through information technology platforms have emerged as a good substitute. In this paper, we attempt to fill the gap by utilizing cellphone-based hourly flow data from a major local telecommunications service provider, combined with detailed city-level employment data, to investigate how mobility responded to lockdown policies in the Philippines.

Our study focuses on work-related mobility encompassing commuting and business trips for four reasons. First, work-related mobility accounts for a majority share of daily travel and is the primary target of many NPIs. Assessing how it has changed in response to lockdown policies can inform us of the effectiveness of the policies to a large extent. Second, the lockdown policies are designed to treat industries differently. Combining detailed spatial distribution of industries and firms of various sizes with work-related mobility, we can learn whether the differential treatments have worked or channels through which lockdown affected mobility. Third, work-related travel is directly associated with employment and production. Focusing on it could give us a better understanding of the adverse impacts of lockdown on the economy. Lastly, responses of work-related and nonwork-related trips to lockdown could be distinct. Not distinguishing between them could lead to uninformative, washed-out measurement of the impacts of lockdown.

In sum, we find that the lockdown has reduced work-related mobility substantially across the country, with reasonably persistent effects. When the stringency of lockdown was lowered, mobility partially recovered. Moreover, there were evident spatial heterogeneities in the effects of lockdown arising from different industrial compositions and firm size compositions across localities. While this implies policies have largely achieved the expected goals, this also reminds us that certain areas and demographic groups require special policy attention, as the impacts of policies vary over space. As far as we know, we are among the first to present evidence and analysis on the topic for a lower-income country.

The rest of the paper is structured as follows. After surveying the emergent literature on NPIs and mobility, we introduce the data used in this study including the proprietary cellphone-based dataset. We then detailed descriptions of the lockdown policies adopted by Philippine local governments over the study period. We examine how mobility responses to lockdowns varied over time and how responses differed by cities’ sectoral composition. We conclude by discussing policy implications of the main findings and drawing possible lessons.

### Literature review

There is a large literature on COVID-19 containment policies, which continues to expand quickly. Here, we focus on surveying studies that examine the proximate response to NPIs: changes in human mobility.

Many studies conducted in the first year of the global pandemic identify large mobility responses to lockdown measures using mobile phone data. For the United States, stay-at-home mandates between January and April 2020 were effective [7]. However, that response was highly context-specific. The effect of NPIs on mobility (measured by average number and length of trips per person) decreased over time and dependent on locations’ average income, industry structure, and demographic composition [8].

A similar picture has emerged from studies of European cities and countries. Based on origin-destination flows captured by mobile phone data in France, mobility fell by 65% due to the nationwide lockdown, with the largest decline in regions that had a high burden of COVID-19 cases, large economically active populations, and heavily employed workers in sectors that either shut down entirely (e.g., restaurants and hospitality) or could work remotely (e.g., financial services) [9]. After quarantine was imposed in Spain in March 2020, mobility as measured by traffic counters and cameras fell by 76% in the city of Santander [10]. The largest decline was in use of public transport, consistent with the finding that volume of commuters at metro stations fell by 80% in Austria following government lockdown [11]. Milder restrictions in Sweden reduced density of commercial and industrial areas by 33% relative to the previous year, according to mobile phone data [12]. Driving, walking, and transit real-time data show that mobility declined gradually in the U.K. in response to government policy, and then stabilized at around 80% of the pre-lockdown norm, significantly reducing COVID-19-related deaths [3].

More recent studies have been able to assess the extent of recovery after removal of restrictions. A spatial discontinuity-based approach shows that Imposition of government lockdowns in Italy lowered mobility by 33%, and that recovery to pre-lockdown mobility was stronger in areas where work-from-home was less prevalent [13]. Mobile phone location data from Japan’s period of COVID-19 “state of emergency” show that significant reductions in travel were achieved without strong government restrictions, but that recovery of mobility after removal of restrictions was incomplete and slow [14].

Turning to cross-country evidence, after analyzing Apple mobility reports of 83 countries from across the income spectrum, controlling for a wide range of factors, walking and driving trip declines were relatively small after imposition of NPIs in countries with high informality, low share of remote work friendly jobs, and low government effectiveness [15]. A multiple-events model using data from 135 countries shows that cancelling public events, imposing restrictions on private gatherings, and school closures had largest effect on reducing COVID-19 incidence, with population mobility as a mediating factor [16]. A Bayesian panel vector autoregression-based analysis using a mobility index based on daily Google visit data from 44 countries reveals that restrictions reduce mobility immediately, reduce cases with a one-week lag, and reduce deaths with a three-week lag [17].

Cross-country regression-based studies also show deteriorating effects of lockdowns over time. Google mobility data from 33 countries show that while the response to restrictions (as measured by a stringency index) was quick, it was not persistent [18]. Strict lockdowns lost their effectiveness (in terms of reducing case load and deaths) after four months based on mobility data from 152 countries, likely due to rising noncompliance [19].

In summary, mobility fell substantially in response to NPIs, but the effects of those lockdowns were heterogeneous by country and typically waned over time. Furthermore, there is within-country evidence that the magnitude of the decline and extent of recovery in mobility depended on locational economic characteristics (notably, the ability of local workforce to work from home). We investigate these issues in the Philippine context after describing the data source.

## Materials and methods

### Data

From one of the major telecommunication companies in the Philippines, we obtain nationwide data of flows of its cellphone users between *barangay* pairs for every hour from January to September 2020. A barangay is the smallest administrative division in the Philippines. One level above barangay are city and municipality with cities being more populated in general. There are 1,634 cities and municipalities consisting of 42,046 barangays in the Philippines by 2019. A problem with the barangay-level flow data is that not every barangay has cell site and the user information in those barangays will be counted for the barangay whose cell site captures it. Aggregating to city and municipality largely mitigates this data bias problem. Moreover, as the primary subnational policymaking body in the Philippines cities and municipalities are of interest from policy perspectives and the other data used in the study—employment by sector and firm size—are only available at city and municipality level. Therefore, we aggregate the barangay-level flows to city and municipality-level flows for this study. For simplicity, we sometimes use city to refer to both.

**Fig 1:**
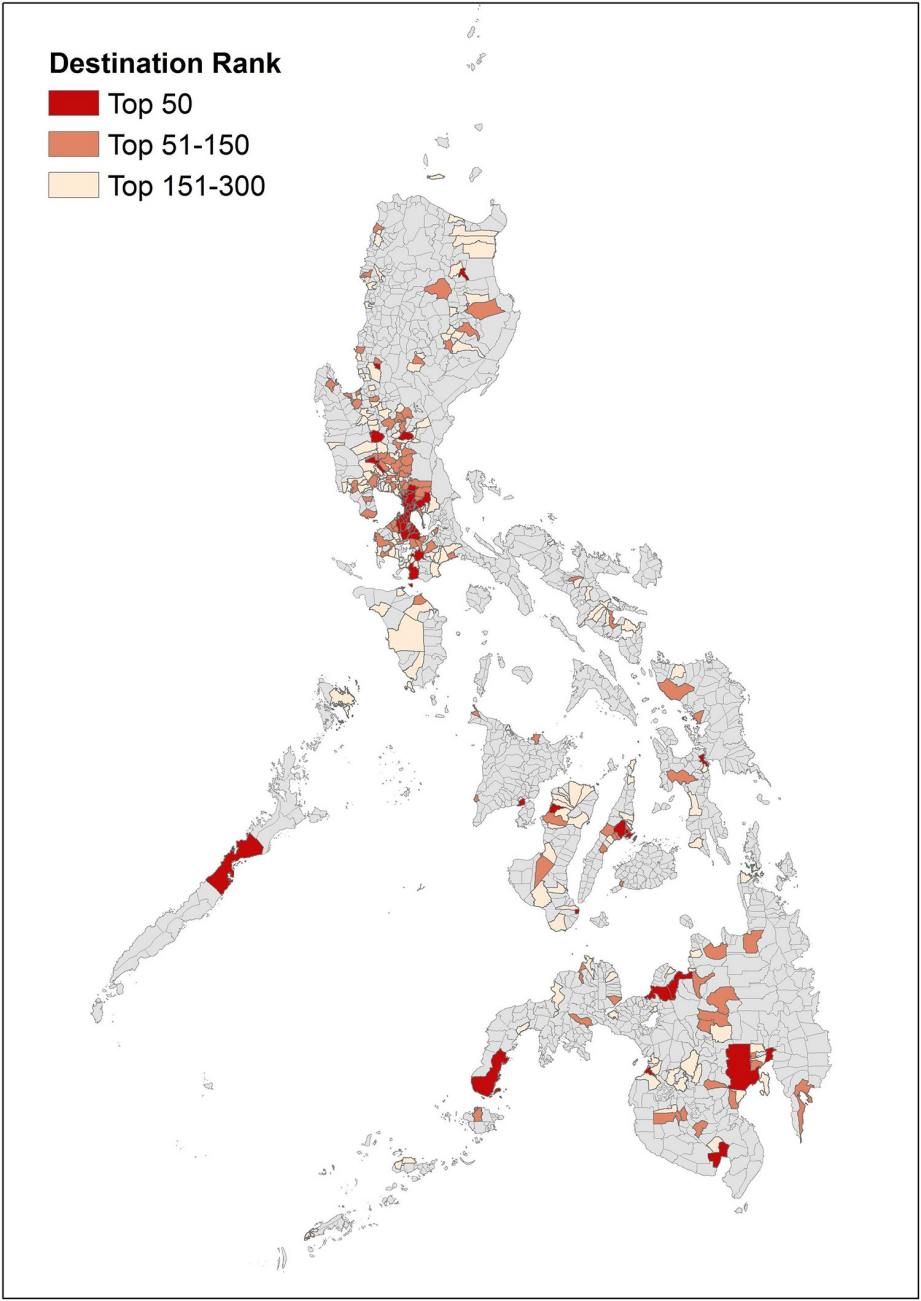
Top 300 morning destination cities and municipalities. Source: Philippine municipality shapefile created by the Philippine Statistics Authority (PSA)in the context of the 2015 population census was retrieved from https://data.humdata.org/dataset/cod-ab-phl. Note: Cities and municipalities are ranked based on the sum of inflows between 4 am and 10 am averaged over weekdays from January 2 to March 13, 2020.

Since we are interested in work-related mobility including commuting and business trips, we select the data based on following criteria. First, we focus on flows taking place from 4 a.m. to 10 a.m. on weekdays. We include 4 a.m. since people started to go to work in the early morning during lockdown to avoid delay at the checkpoints. Second, to focus the analysis on urban areas hosting the majority of national employment, we restrict the sample to the most popular destination cities in terms of pre-lockdown morning trips. Specifically, we calculate sum of hourly flows into each city and municipality between 4 a.m. and 10 a.m. and average it across weekdays from January 2 to March 13, 2020. The 300 cities and municipalities with the highest average inflows are retained as destinations from the total 1,560. These top 300 destination cities and municipalities account for 78% of total morning flows, 57% of population, 86% of employment, and 21% of the area of the country. Fig 1 illustrates where these top 300 are located across the country. Third, we exclude all city pairs with road-based distance exceeding 150 kilometers from the analysis, considering the excluded pairs to be too far apart for regular commuting trips. This leaves us with 1,387 cities and municipalities as origins and more than 39,000 origin-destination (OD) pairs.

We sum flows over the six morning hours and then average them across workdays for each week. The unit of observation is thus city-level OD pair per week. There are 40 weeks between January and September. Lockdown measures have been imposed in most areas of the Philippines since the 12th week of 2020 (i.e., March 16 to 20). Upon checking, however, the hourly profiles of the nine weeks in May and June show patterns distinct from those of other weeks (S1 Fig). Absent a clear explanation for this irregularity, we exclude data of these weeks from analysis. Thus, the final dataset we work with covers 31 weeks, 20 of which coincided with the pandemic.

To validate whether the cellphone-based OD flows are a good measure of work-related mobility, we correlate them with the commuting flows between cities and municipalities derived from microdata of 2010 Census of Population and Housing, which is the latest available Philippine census. The correlation coefficient between census-based commuting flows and average cellphone-based morning flows over all pre-COVID-19 weeks (weeks 1–11) is around 0.95, suggesting the latter is a valid work-related mobility measure.

Table 1A reports summary statistics of flows by week. Positive flows are observed for about 34,000 to 35,000 pairs of cities before the 12th week. The average flows ranged from 2,800 to 3,400 and median flows from 15 to 20 in the eleven pre-lockdown weeks. In the 12th week, the number of observed OD pairs dropped by 11% to 30,838, followed by another 11% drop in the 13th week as the pandemic unfolded. The median flow decreased from 18 in the preceding weeks to 11 in the 12th week and further down to 7 in the 13th week although the mean flows declined at a slower pace. Over the period covered, we see the flow statistics including mean, mean, and total remain in the trough until the 27th week and started to rebound from the 28th week when there was a nationwide relaxation of lockdown. Up till the 40th week, however, the total flows had only recovered to less than 90% of the pre-lockdown level. The patterns of flows and how they respond to different lockdown stringencies will be further explored and discussed in the following sections of the paper.

**Table 1A:** Summary statistics of morning flows by week.

Week	Observations	Mean	SD	Min	Median	Max	Total
1	33,954	3,190	57,431	1	15	5,664,627	108,321,048
2	35,186	3,378	66,768	1	20	7,037,750	118,869,752
3	34,289	3,252	62,899	1	17	6,213,362	111,507,556
4	34,794	3,350	67,365	1	19	6,811,825	116,576,438
5	34,938	3,233	64,694	1	19	6,603,484	112,949,349
6	34,697	3,090	60,498	1	18	6,127,628	107,206,550
7	34,732	3,072	61,589	1	18	6,464,194	106,708,683
8	34,748	3,022	61,169	1	18	6,385,326	105,000,276
9	33,807	3,053	59,745	1	16	6,175,062	103,208,129
10	34,662	2,886	56,009	1	18	5,628,076	100,047,743
11	34,924	2,772	52,062	1	18	5,194,160	96,798,810
12	30,838	2,733	45,395	1	11	3,849,394	84,271,812
13	27,416	2,694	42,101	1	7	3,365,286	73,859,958
14	26,705	2,615	40,119	1	7	3,232,383	69,821,185
15	25,022	2,881	42,949	1	6	3,417,396	72,080,578
16	28,041	2,620	41,183	1	8	3,422,339	73,471,670
17	28,016	2,515	39,268	1	7	3,235,731	70,473,324
27	28,363	1,932	30,595	1	9	2,617,903	54,788,292
28	33,490	2,595	45,650	1	14	4,298,624	86,909,641
29	33,770	2,669	47,001	1	14	4,484,620	90,148,082
30	33,474	2,557	44,549	1	14	4,157,871	85,597,684
31	32,721	2,776	48,429	1	13	4,650,929	90,833,347
32	32,377	2,760	47,594	1	13	4,336,184	89,344,579
33	32,461	2,802	48,141	1	13	4,367,758	90,959,328
34	31,966	2,873	49,375	1	12	4,537,778	91,840,516
35	33,177	2,776	48,909	1	14	4,739,307	92,099,492
36	32,103	2,765	47,972	1	12	4,539,652	88,769,274
37	33,234	2,623	46,078	1	14	4,427,404	87,161,264
38	33,155	2,602	45,570	1	14	4,296,799	86,268,066
39	33,425	2,620	46,674	1	14	4,380,945	87,573,912
40	31,470	2,762	47,474	1	11	4,293,096	86,916,676

SD = standard deviation.

Notes: 1. Morning refers to 4 a.m. to 10 a.m.

2. Weekly data is average of daily morning flows over weekdays.

3. Each observation is weekly average flow between a pair of cities/municipalities with one of the top 300 cities/municipalities as destination.

Source: Authors’ estimation.

Compared to Google Community Mobility Report (CMR) data which provides a daily measure available at more aggregate region level and for eight metropolitan areas in the Philippines, our mobility data covers the whole country with higher time frequency as well as space granularity. On the other hand, Google CMR data distinguishes mobility by location type, which may allow more precise identification of work-related mobility.

List of Establishments 2018 (LE2018 hereafter) data is the second main data source used in this study. The LE2018 obtained from Philippine Statistics Authority (PSA) contains total employment at city/municipality level for metro Manila and at province level for the rest of the country and number of firms at city and municipality level by size category and sector. There are four size categories, which are micro with 1–9 employees, small 10–99, medium 100–199 and large 200 and over. To derive employment at city and municipality level outside metro Manila, we first calculate total number of firms by size and sector for each province. Then, the total employment is divided by total number of firms to obtain mean employment by size and sector at province level. Assuming that the constituent cities and municipalities have the same mean employment for each combination of size and sector as their province does, the mean employment is multiplied with number of firms to reach total employment by size and sector at city and municipality level.

To focus the analysis, we create two firm size groups by lumping the micro and small firms together and medium and large firms together. The sectors are also grouped into seven categories based on similarity, share in the economy, and ease of working from home of the industries. For instance, one sector category encompasses power, utilities and construction industries, another trade and transport industries, and a third hospitality and recreation industries. Other tertiary industries are allocated into work-from-home (WFH) -friendly and non-WFH-friendly categories, respectively, based on 3-digit industry codes and the classification system for skilled scalable services [20], with some discretionary adjustments for the Philippines in the context of the pandemic (e.g., classifying call center jobs and teaching as WFH-friendly).

The literature often quantifies the WFH propensity by occupation [21]. In the absence of spatial distribution data of occupations, industry-based categorization likely provides a second-best measurement of how different cities are amenable to WFH. Moreover, from a public policy perspective, understanding how cities with different industrial compositions respond to lockdown measures differently is important as it would be easier for policy interventions to be targeted at industries than at occupations.

Table 1B shows that employment by micro and small firms accounts for 73% of total employment across the top 300 cities and municipalities on average, with individual cities’ shares ranging between 13% to 100%. Medium and large firms hire between 0% to 87% across cities, with the average a little more than one quarter.

**Table 1B:** Summary statistics of city employment shares for top 300 destination cities and municipalities.

Classification	Category	Observations	Mean	SD	Min	Median	Max
**Firm size**	Small and micro	300	0.732	0.202	0.125	0.772	1.000
	Medium and large	300	0.268	0.202	0.000	0.228	0.875
**Sector**	Manufacturing	300	0.179	0.155	0.017	0.121	0.820
	Primary	300	0.040	0.074	0.000	0.013	0.489
	Power, utilities, and construction	300	0.039	0.036	0.000	0.028	0.229
	Trade and transport	300	0.351	0.113	0.047	0.353	0.748
	Hospitality and recreation	300	0.132	0.070	0.016	0.124	0.599
	WFH-friendly tertiary	300	0.133	0.071	0.005	0.121	0.550
	Non-WFH-friendly tertiary	300	0.126	0.070	0.012	0.113	0.513

SD = standard deviation; WFH = work-from-home.

Notes: 1. Total employment is divided by total number of firms for each firm size and sector combination to get mean employment by firm size and sector in each province.

2. The city-level total employment equals the product of provincial mean employment and city-level number of firms in each firm size and sector combination.

Source: Philippine Statistics Authority. List of Establishments 2018. Manila.

Philippine cities vary considerably in terms of industrial composition. On average, trade and transport combined is the largest sector category, followed by manufacturing, hospitality and recreation, and WFH-friendly tertiary. On one end of the sector distribution spectrum, manufacturing employment accounts for 82% of total employment. On the other end, trade and transport account for 75%. There are other cities dominated by hospitality and recreation sectors, WFH-friendly tertiary sectors or non-WFH-friendly tertiary sectors, which are likely to respond to mobility restrictions differently.

Other data used in the study are lockdown policies from the website of the Philippines Official Gazette and daily COVID-19 infection cases from the website of Department of Health. Both are processed to the city- and municipality-week level.

### COVID-19 lockdown policies in the philippines

Faced with the threat of COVID-19 in the Philippines, the government introduced “community quarantine” as a restriction policy to contain the rapid transmission of the virus. The policy has four levels: enhanced community quarantine (ECQ), modified enhanced community quarantine (MECQ), general community quarantine (GCQ), and modified general community quarantine (MGCQ). Among levels, ECQ is the most stringent while MGCQ is the most lenient.

According to [22], the mobility in cities and municipalities placed under ECQ is generally restricted, with public transportation suspended. Only those individuals accessing basic goods and services and workers in industries permitted to operate are authorized to leave their residences. Essential industries, particularly hospitals and front-line health services, manufacturers of essential goods, agriculture, forestry, fishing, and logistics and delivery service providers of essential goods, are allowed to operate with full on-site capacity. Industries providing essential retail trade and services, food preparation (for take-out and delivery), and media outlets are also allowed but should operate at 50% of maximum workforce on-site and the remaining through work-from-home or other flexible work arrangements.

Non-essential businesses are permitted with skeleton on-site capacity during ECQ. This includes other health-related, financial institutions, water facilities and sanitary services, energy sector, information technology and telecommunication companies, and aviation and maritime industries. In addition, skeleton arrangement is also enforced to other manufacturing companies, business process outsourcing (BPO), export-oriented, mining and quarrying businesses, construction companies of priority projects, repair and maintenance establishments, property renting businesses, employment agencies for permitted sectors, and other essential services. Similarly, government offices function with skeleton staff and through alternative work arrangements. On the other hand, hospitality industries are not allowed to operate, except those accommodation establishments accredited to cater guests and clients for legitimate purposes under a state of public health emergency but should only provide basic services through skeleton employees. Also, all recreation industries are prohibited to operate during ECQ.

While MECQ is the less stringent variation of ECQ, the population is still required to stay at home. Public transportation remains unavailable, and travel is limited to authorized persons going to work or acquiring essential goods and services. However, more industries are permitted to operate with full on-site capacity. The additional industries include the media, BPOs, export-oriented establishments, e-commerce companies, property renting businesses, employment agencies for permitted sectors, and housing services. All other industries previously operating with skeleton workers, including establishments providing professional, scientific, and technical services, are permitted with 50% personnel. But government offices continue to operate with minimum capacity under MECQ. Hospitality and leisure establishments follow the same restriction as in ECQ.

For cities and municipalities under GCQ, public transportation is permitted at reduced capacity with strict health protocols. But the guideline on the movement of individuals is the same as for the more stringent measures. For industries, full operational capacity is extended to energy, water, other utilities, health-related, information technology, and telecommunications industries. Likewise, mining, quarrying, other manufacturing, repair and maintenance, and housing and office services are permitted at operational capacity between 50% and 100%. Under GCQ, other permitted industries must follow the same capacity and work arrangements as under MECQ. The same limitation is imposed on hospitality and leisure industries as for previous measures.

Relaxed restriction is imposed during MGCQ. Those government and private offices and industries previously allowed can operate between 50% and 100% capacity with an option of flexible working. Hospitality and recreational establishments are also allowed at a maximum of 50% capacity. However, for accommodation businesses, only those accredited may operate. Furthermore, all forms of transportation are generally allowed but should follow health protocols. Physical non-contact activities are also permitted within place of residence under MGCQ.

**Fig 2:**
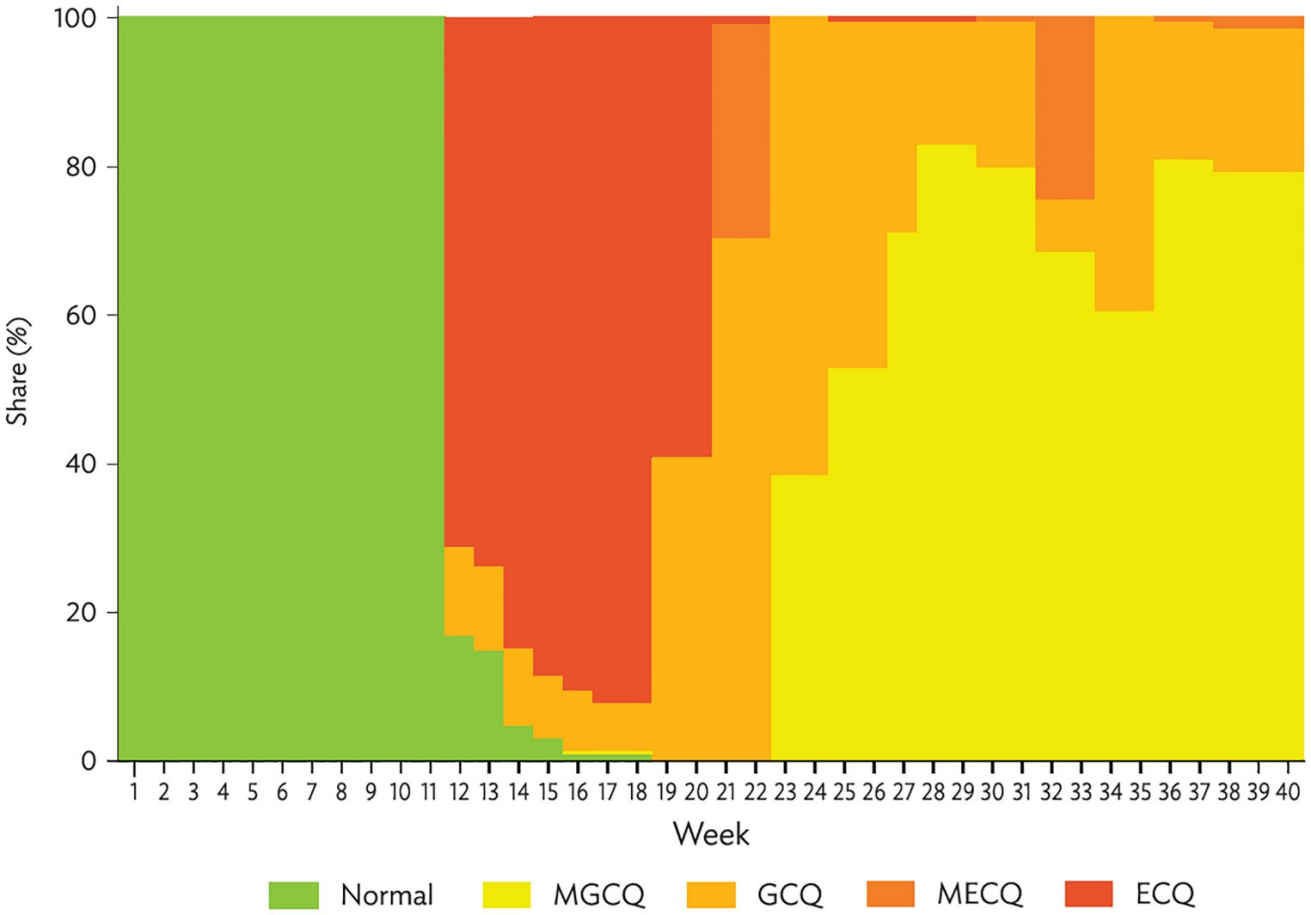
Share of community quarantine measures by week among top 300 destinations. ECQ = enhanced community quarantine; GCQ = general community quarantine; MECQ = modified enhanced community quarantine; MGCQ = modified general community quarantine. Note: Lockdown policies data is obtained from the Government of the Philippines. *Official Gazette*. https://www.officialgazette.gov.ph/section/laws/other-issuances/inter-agency-task-force-for-the-management-ofemerging-infectious-diseases-resolutions/. Source: Authors’ calculations.

In Fig 2, we present the share of the top 300 city and municipality destinations under the various stringency measures by week. Although COVID-19 was first detected in the Philippines in the last week of January 2020 (week 5), the lockdown measures were imposed beginning in the third week of March 2020 (week 12). As shown in the chart, the country was in normal state (i.e., unrestricted) from weeks 1 to 11 before 71% of the areas were placed under ECQ in week 12. By week 18 (last week of April) ECQ covered 92% of the top 300 cities and municipalities. There was an easing of lockdown to GCQ in some areas during the first half of May (weeks 19 and 20). However, 60% remained under ECQ, particularly Metro Manila and surrounding provinces, Metro Cebu, and Metro Davao due to prevailing high-risk COVID-19 infections. Subsequently, 70% total areas shifted to GCQ in the second half, whereas Metro Manila and other high-risk areas moved to MECQ.

Many cities and municipalities gradually transitioned to less stringent measures in the following months. In the first week of June (week 23) 61% were under GCQ, and by the end of July (week 31) 80% had eased to MGCQ. At the same time, Metro Manila and nearby provinces and other metropolitan areas shifted to GCQ. However, MECQ was reimposed in Metro Manila and neighboring provinces for two weeks in August (weeks 32 and 33). This was after medical frontliners appealed to the government for a break to prevent the collapse of the health care system amidst the continuous surge in COVID-19 cases in those areas. Since then, Metro Manila and neighboring areas reverted to GCQ, with most of the country in MGCQ.

## Results

### Time profile of mobility response to lockdown policies

To assess how mobility evolved over the first six months of the COVID-19 pandemic in the 300 most active Philippine cities, we regressed change in OD flows relative to the pre-lockdown baseline (the first 9 weeks of 2020) on weeks elapsed since first national imposition of ECQ, with destination city fixed effects (*α*_*d*_) and standard errors clustered at the destination city-week level (*ε*_*od*,*w*_):

$$\%\;\;change\;in\;flows\;relative\;to\;baseline_{od,w}=\alpha_{d}+\overset{30}{\underset{x=0}{\sum }}\beta_{x}{\left[ECQ\;\;imposed\;x\;weeks\;ago\right]}_{w}+\varepsilon_{od,w}$$

**Fig 3:**
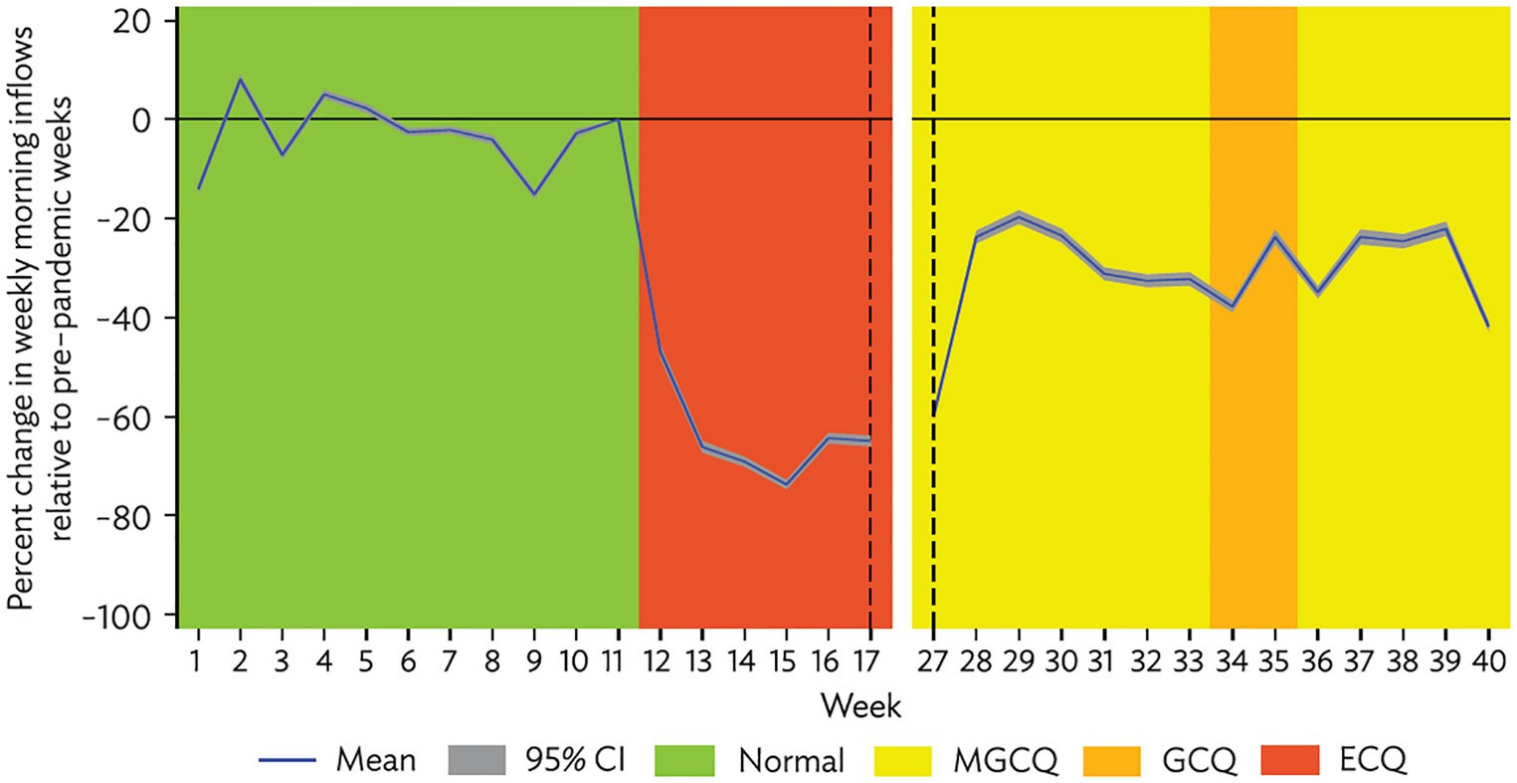
National mobility response to imposition of enhanced community quarantine. CI = confidence interval; ECQ = enhanced community quarantine; GCQ = general community quarantine; MECQ = modified enhanced community quarantine; MGCQ = modified general community quarantine. Note: Shading reflects the weekly most common form of community quarantine (at the origin level). Source: Authors’ calculations.

**Fig 4:**
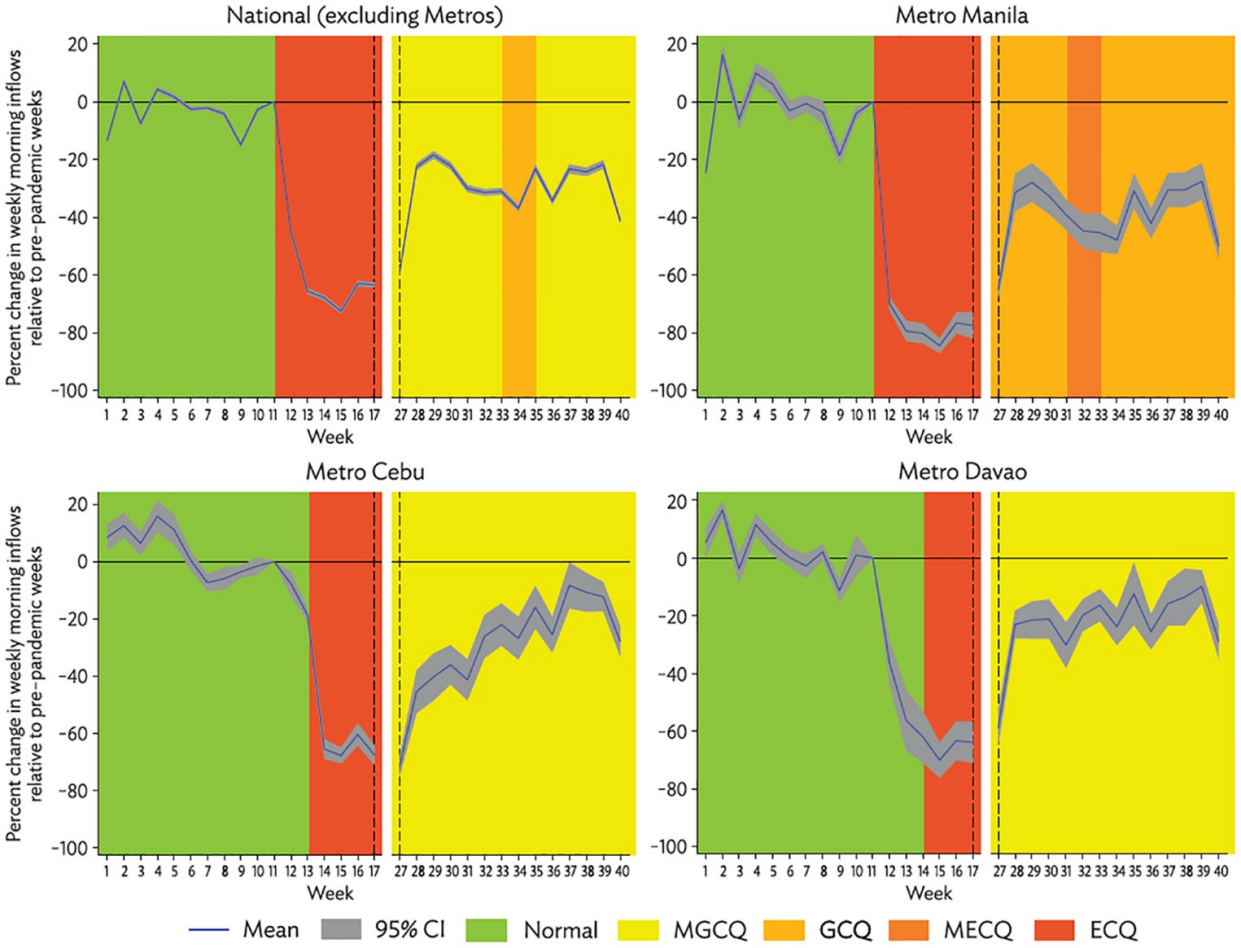
Subnational mobility response to imposition of enhanced community quarantine. CI = confidence interval; ECQ = enhanced community quarantine; GCQ = general community quarantine; MECQ = modified enhanced community quarantine; MGCQ = modified general community quarantine. Source: Authors’ calculations.

The resulting impulse response regression coefficients (*β*_*x*_) are shown in Fig 3 for the country (change in flows based on 1,001,955 OD pairs), and then in Fig 4 separately for non-metro cities (871,932 OD pairs), Metro Manila (104,681 OD pairs), Metro Cebu (13,444 OD pairs) and Metro Davao (11,989 OD pairs).

Mobility relative to the pre-lockdown norm declined precipitously in the month following ECQ imposition, reaching a low of 26% in the fourth week after ECQ before recovering somewhat and stabilizing at around 70% of the pre-lockdown level of flows when most of the country was under MGCQ in August and September of 2020.

The mobility response was most pronounced for Metro Manila, where flows fell to as low as 15% of the pre-lockdown norm. The recovery in the second half of 2020 was also weakest for Metro Manila, which remained in GCQ while the rest of the country was largely in MGCQ. The response was similar among non-metro cities, Metro Cebu, and Metro Davao, where the low was only around 30% of the pre-lockdown norm and recovery to previous levels of mobility in the second half of 2020 was stronger than for Metro Manila (though there was a notable dip in flows in the last week of our data).

While the picture is interrupted due to the data issues for May and June noted in the data section, this analysis shows that urban mobility in the Philippines was highly responsive to community quarantines and appears to have tracked stringency levels in the expected manner, with the largest declines experienced during ECQ and recovery experienced during MGCQ. Overall, the quarantine policies have been effective persistently, which should have had significant impacts on the economy as well as containing COVID spread.

### Geographic profile of mobility response to lockdown

#### Empirical models and conjectures

It is expected that there is significant variation across cities in mobility responses to lockdown measures based on the nature of their pre-COVID-19 economic activity. To assess this heterogeneity, we focus on two aspects of workplace cities: industrial composition and firm size composition. We focus on these because lockdown policies treat or affect industries differently as necessity, human contact intensity, and remote workability vary across industries and lockdown enforcement cost could be closely related to firm size, which is related to formality.

We employ the following gravity regression to examine this:

$$\begin{array}{ll} y_{odt} & =\alpha_{0}+\sum_{l}\gamma_{l}Z_{dt}^{l}+\sum_{l}\sum_{e}\gamma_{el}X_{ed}Z_{dt}^{l}+\beta_{1}dist_{od}+\beta_{2}nwd_{t}+\beta_{3}stricter_{odt}\\ & +\beta_{4}looser_{odt}+\beta_{5}cases_{dt-1}+\beta_{6}cases_{ot-1}+\beta_{7}cases_{dt-2}+\beta_{8}cases_{ot-2}+\theta_{o}+\theta_{d}+\varepsilon_{odt} \end{array}$$

where *y*_*odt*_ is the ratio of average flow between origin city *o* and destination city *d* of week *t* to the average pre-lockdown flow, $Z_{dt}^{l}$ is a dummy equal to 1 if city *d* adopted lockdown measure *l* in week *t* and 0 otherwise, *X*_*ed*_ is the share of employment in industrial category *e* or share of employment in medium and large firms of city *d*. Here *l* = *ECQ* + *MECQ*, *GCQ or MGCQ* as we combine ECQ and MECQ for simplicity since MECQ accounts for a very small share in the sample. Pre-lockdown weeks are the omitted category. The parameters *γ*_*l*_’s measure benchmark effects of lockdown policies on mobility without considering differences in destination city’s industrial characteristics. Of main interest are *γ*_*el*_’s, which measure the heterogenous effects of lockdown varying by city industrial characteristics. Specifically, we expect that: (i) cities with larger shares in tertiary industries would have experienced a more pronounced decrease in mobility while those with more employment in manufacturing and trade and transport industries experienced smaller declines; (ii) when lockdown stringency was reduced, more mobility rebound would have been observed in cities with more non-WFH-friendly industries as compared to those with more WFH-friendly industries; and (iii) mobility would have declined more in cities with a greater share of workers in medium and large firms.

To obtain consistent estimates of *γ*_*l*_’s and *γ*_*el*_’s, we control for a set of variables including the road-based distance between the origin and destination (*dist*_*od*_), number of working days in each week (*nwd*_*t*_), whether the origin city’s lockdown was more or less stringent than that of destination city (*stricter*_*odt*_ and *looser*_*odt*_), numbers of cases in destination and origin cities in the past two weeks (*cases*_*dt*−1_, *cases*_*ot*−1_, *cases*_*dt*−2_, and *cases*_*ot*−2_) as well as origin and destination dummies (*θ*_*o*_ and *θ*_*d*_). While the pandemic spread and lockdown rollout were inextricably linked, controlling for lagged local COVID-19 cases separates the response to lockdown policy from the response to perceived risk of infection as much as the data allow.

The lockdown stringency was adjusted by the government mainly based on the pandemic situation. While it is unlikely to have reverse causality whereby the observed mobility led to change in lockdown policies, the omitted variables such as utilization of ICU beds could be correlated with both mobility and lockdown stringency. To address this, we include lagged mobility variable as regressor in a second specification. Furthermore, we replace separate origin and destination dummies with origin-destination pair dummies in a third specification. Results reported below show estimates from these three specifications. Stable coefficient estimates would suggest that the model is not subject to severe endogeneity arising from omitted variables.

#### Baseline results

Tables 2A and 2B report estimates of *γ*_*l*_’s and *γ*_*el*_’s with *X*_*ed*_ being share of employment in industrial category and share in medium and large firms, respectively. Other coefficients are suppressed to save space. Across three model specifications, the estimates are qualitatively similar while some diminish slightly after controlling for lagged mobility. Hence, we focus on results from the third specification hereafter since it minimizes the potential endogeneity and has the highest adjusted R-squared.

**Table 2A:** Effects of lockdown measures by destination city industrial composition (baseline).

Variables	Model 1		Model 2		Model 3	
	β	SE	β	SE	β	SE
Benchmark effects						
ECQ+MECQ	-0.599***	(0.020)	-0.459***	(0.018)	-0.474***	(0.018)
GCQ	-0.284***	(0.023)	-0.153***	(0.020)	-0.165***	(0.020)
MGCQ	-0.281***	(0.020)	-0.146***	(0.017)	-0.162***	(0.017)
ECQ+MECQ dummy × Employment share of industry category						
Primary	0.393***	(0.071)	0.315***	(0.058)	0.320***	(0.060)
Power, utilities, construction	-0.441***	(0.108)	-0.335***	(0.085)	-0.366***	(0.087)
Trade, transport	0.316***	(0.038)	0.212***	(0.030)	0.241***	(0.031)
Hospitality, recreation	-0.126	(0.066)	-0.107	(0.056)	-0.121*	(0.057)
WFH-friendly tertiary	-0.342***	(0.052)	-0.249***	(0.043)	-0.276***	(0.044)
Non-WFH-friendly tertiary	-0.345***	(0.065)	-0.202***	(0.054)	-0.247***	(0.055)
GCQ dummy × Employment share of industry category						
Primary	0.159	(0.093)	0.040	(0.079)	0.050	(0.080)
Power, utilities, construction	-0.376**	(0.127)	-0.323**	(0.108)	-0.344**	(0.110)
Trade, transport	0.058	(0.048)	0.015	(0.041)	0.024	(0.041)
Hospitality, recreation	-0.126	(0.088)	-0.109	(0.073)	-0.125	(0.074)
WFH-friendly tertiary	-0.511***	(0.052)	-0.395***	(0.046)	-0.420***	(0.046)
Non-WFH-friendly tertiary	-0.297***	(0.077)	-0.109	(0.066)	-0.130	(0.066)
MGCQ dummy × Employment share of industry category						
Primary	0.264***	(0.069)	0.169**	(0.058)	0.186**	(0.060)
Power, utilities, construction	-0.246*	(0.098)	-0.143	(0.083)	-0.157	(0.083)
Trade, transport	0.233***	(0.035)	0.129***	(0.031)	0.146***	(0.031)
Hospitality, recreation	-0.097	(0.072)	-0.094	(0.063)	-0.095	(0.064)
WFH-friendly tertiary	-0.534***	(0.059)	-0.403***	(0.052)	-0.417***	(0.052)
Non-WFH-friendly tertiary	-0.168**	(0.056)	-0.116*	(0.048)	-0.128**	(0.048)
Origin and destination dummies	Yes		Yes		No	
AR(1)	No		Yes		Yes	
Origin-destination dummy	No		No		Yes	
Observations	931,233		902,924		902,924	
Adjusted R-squared	0.265		0.322		0.386	

AR(1) = autoregressive term, lag 1; ECQ = enhanced community quarantine; GCQ = general community quarantine; MECQ = modified enhanced community quarantine; MGCQ = modified general community quarantine.

Notes: 1. Other regressors not shown in the table include the road-based distance between the origin and destination (except model 3), number of working days in each week, whether the origin city’s lockdown was more or less stringent than that of destination city, numbers of cases in destination and origin cities in the past two weeks.

2. Standard errors are clustered at the destination-week level.

3. ***, **, * indicate significant coefficients at 1%, 5%, 10%, respectively.

Source: Authors’ calculations.

**Table 2B:** Effects of lockdown measures by destination city firm size composition (baseline).

Variables	Models		
	1	2	3
Benchmark effects			
ECQ+MECQ	-0.534***	-0.417***	-0.427***
	(0.007)	(0.009)	(0.008)
GCQ	-0.342***	-0.201***	-0.215***
	(0.010)	(0.010)	(0.010)
MGCQ	-0.256***	-0.153***	-0.163***
	(0.006)	(0.007)	(0.007)
ECQ+MECQ dummy × Employment share of medium and large firms	-0.241***	-0.162***	-0.186***
	(0.020)	(0.016)	(0.017)
GCQ dummy × Employment share of medium and large firms	-0.161***	-0.107***	-0.119***
	(0.026)	(0.021)	(0.021)
MGCQ dummy × Employment share of medium and large firms	-0.169***	-0.096***	-0.107***
	(0.019)	(0.016)	(0.016)
Origin and destination dummies	Yes	Yes	No
AR(1)	No	Yes	Yes
Origin-destination dummy	No	No	Yes
Observations	931,233	902,924	902,924
Adjusted R-squared	0.262	0.321	0.384

AR(1) = autoregressive term, lag 1; ECQ = enhanced community quarantine; GCQ = general community quarantine; MECQ = modified enhanced community quarantine; MGCQ = modified general community quarantine; SE = standard errors; WFH = work-from-home.

Notes: 1. Other regressors not shown in the table include the road-based distance between the origin and destination (except model 3), number of working days in each week, whether the origin city’s lockdown was more or less stringent than that of destination city, numbers of cases in destination and origin cities in the past two weeks.

2. Standard errors are clustered at the destination-week level.

3. ***, **, * indicate significant coefficients at 1%, 5%, 10%, respectively.

Source: Authors’ calculations.

First, Table 2A shows that the benchmark effects decline from 47% reduction in mobility under ECQ+MECQ to around 16% under GCQ or MGCQ. This confirms again that ECQ+MECQ as the most stringent lockdown policy had the largest impacts on mobility. It is notable that GCQ and MGCQ had almost the same effects if not accounting for industrial differences across cities.

The coefficients of interaction terms suggest that cities with more employment in power, utilities and construction, hospitality and recreation, WFH-friendly or non-WFH-friendly tertiary industries have experienced greater declines in mobility, regardless of lockdown stringency. For instance, should the employment share of WFH-friendly tertiary sectors increase by 10 percentage points in a destination city, the expected morning inflows would decrease by additional 2.8%, 4.2% and 4.2% when the city moves from normal period to the respective ECQ+MECQ, GCQ, and MGCQ scenarios. Note that the estimates measure marginal effects of employment in WFH-friendly sectors on top of benchmark effect, so they could be larger under less stringent lockdown. The overall reduction in mobility is still bigger under more stringent lockdown.

In contrast, cities with larger employment in trade and transport industries have in general experienced smaller declines in the mobility. Compared with the benchmark, a 10 percentage points increase in employment share of trade and transport industries would predict 2.4% less decrease in morning flows into the city under ECQ+MECQ. Primary sector employment had similar dampening effects on the lockdown measures.

**Fig 5:**
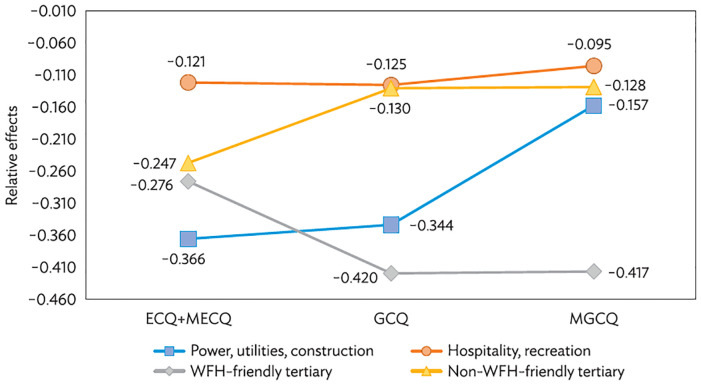
Differential effects of lockdown measures on cities of different industrial corporation. ECQ = enhanced community quarantine; GCQ = general community quarantine; MECQ = modified enhanced community quarantine; MGCQ = modified general community quarantine; WFH = work-from-home. Source: Authors’ calculations (see column “Model 3” in Table 2A).

Furthermore, we see varying effects of industrial shares under different lockdown measures, which are in line with our second conjecture. Fig 5 plots the estimated *γ*_*el*_’s for four industrial categories with additional adverse effects from lockdowns. With implementation of GCQ and MGCQ, the decrease in mobility (relative to the benchmark) in cities with larger power, utilities and construction and non-WFH-friendly tertiary industries shrank towards the benchmark suggesting considerable job/business rebound in these sectors.

In contrast, the relative effects of WFH-friendly sectors become larger in magnitude under GCQ and MGCQ. A simple calculation shows that moving from ECQ to GCQ, the benchmark mobility will increase from 53% (= 100% − 47%) to 83% (= 100% − 17%) of the pre-COVID-19 level. Nonetheless, for a city with 20% employment in WFH-friendly sectors, the mobility will shift from 47% (= 100% − 47% − 28% × 20%) of pre-COVID-19 level under ECQ to 75% (= 100% − 17% − 42% × 20%) only under GCQ. Hence, a large share of employment in WFH-friendly sectors could help keep mobility low even when the lockdown policy is not highly restrictive.

Hospitality and recreation industries belong to the non-WFH-friendly tertiary category. However, they are separately analyzed because they were specially targeted by the lockdown policies. Only under the least stringent MGCQ were they allowed to operate with maximum of 50% capacity. These industries did perform differently from other non-WFH-friendly industries. Under the most stringent ECQ, the relative effect of lockdown for employment share in hospitality and recreation is just half of that for non-WFH-friendly industries. Driven by job recovery in non-WFH-friendly industries, the two estimates converge under GCQ. Only with implementation of MGCQ does the mobility due to hospitality and recreation rebound moderately, as suggested by the estimate changing from -0.121 to -0.095. The results observed here may be because hospitality- and recreation-related mobility is more sensitive to the threat of COVID-19 (proxied by numbers of new cases in the past two weeks) than to government regulations. To avoid infection, people self-regulated on entertainment activities such as dining out and partying more than they did for other, less discretionary economic activities. As a support, we do find that the coefficients of interactions between lockdown and share of hospitality and recreation are larger in magnitude with uniform statistical significance in regressions without lagged number of infection cases.

In Table 2B, we show that cities with higher employment share in large and medium firms experienced a greater decline in morning inflows. An increase of 10 percentage points in employment share in large and medium firms led to 1.9% decrease in mobility on top of benchmark reduction when ECQ/MECQ was in place. Echoing the findings in the literature [15], this may be related to the lockdown enforcement cost or tendency to comply. It is easier for the government to monitor and enforce lockdown measures with larger employers and becomes much costlier when the firms are small and likely informal as well. It is also possible that larger firms chose to be more compliant with the lockdown policies. The differential effects, however, decreased considerably when the lockdown was relaxed to GCQ and MGCQ. This is expected as gaps in enforcement costs or compliance tendency closed under less stringent lockdown.

#### Robustness checks

We consider three sensitivity analyses to test robustness of the results presented above. First, there was large number of short-distance trips in the data, for instance those found moving within a barangay. A considerable share of these trips may not be related to work, even if they took place in the peak hours. Thus, they may have different patterns in terms of responding to the lockdown measures. We excluded within-barangay flows, which account for 40.0% of total flows, to examine whether the baseline results are retained among the remaining flows. The estimates are reported in Tables 3A and 3B.

**Table 3A:** Effects of lockdown measures by destination city industrial composition (excluding within-barangay flows).

Variables	Model 1		Model 2		Model 3	
	β	SE	β	SE	β	SE
Benchmark effects						
ECQ+MECQ	-0.599***	(0.020)	-0.460***	(0.018)	-0.476***	(0.018)
GCQ	-0.284***	(0.023)	-0.153***	(0.020)	-0.166***	(0.020)
MGCQ	-0.279***	(0.020)	-0.146***	(0.017)	-0.162***	(0.017)
ECQ+MECQ dummy × Employment share of industry category						
Primary	0.386***	(0.071)	0.311***	(0.058)	0.316***	(0.060)
Power, utilities, construction	-0.443***	(0.109)	-0.336***	(0.085)	-0.367***	(0.087)
Trade, transport	0.316***	(0.038)	0.212***	(0.030)	0.241***	(0.031)
Hospitality, recreation	-0.131*	(0.067)	-0.110*	(0.056)	-0.125*	(0.057)
WFH-friendly tertiary	-0.342***	(0.052)	-0.249***	(0.043)	-0.276***	(0.044)
Non-WFH-friendly tertiary	-0.342***	(0.065)	-0.200***	(0.054)	-0.245***	(0.055)
GCQ dummy × Employment share of industry category						
Primary	0.155	(0.093)	0.037	(0.079)	0.047	(0.080)
Power, utilities, construction	-0.378**	(0.126)	-0.324**	(0.108)	-0.345**	(0.110)
Trade, transport	0.057	(0.048)	0.014	(0.041)	0.022	(0.041)
Hospitality, recreation	-0.129	(0.088)	-0.111	(0.073)	-0.126	(0.074)
WFH-friendly tertiary	-0.511***	(0.052)	-0.395***	(0.046)	-0.420***	(0.046)
Non-WFH-friendly tertiary	-0.295***	(0.077)	-0.108	(0.066)	-0.129	(0.066)
MGCQ dummy × Employment share of industry category						
Primary	0.259***	(0.069)	0.165**	(0.058)	0.183**	(0.060)
Power, utilities, construction	-0.249*	(0.098)	-0.146	(0.083)	-0.159	(0.083)
Trade, transport	0.231***	(0.035)	0.127***	(0.031)	0.145***	(0.031)
Hospitality, recreation	-0.101	(0.072)	-0.097	(0.063)	-0.098	(0.064)
WFH-friendly tertiary	-0.537***	(0.059)	-0.405***	(0.052)	-0.419***	(0.052)
Non-WFH-friendly tertiary	-0.169**	(0.056)	-0.117*	(0.048)	-0.128**	(0.049)
Origin and destination dummies	Yes		Yes		No	
AR(1)	No		Yes		Yes	
Origin-destination dummy	No		No		Yes	
Observations	931,233		902,924		902,924	
Adjusted R-squared	0.266		0.323		0.386	

AR(1) = autoregressive term, lag 1; ECQ = enhanced community quarantine; GCQ = general community quarantine; MECQ = modified enhanced community quarantine; MGCQ = modified general community quarantine; SE = standard errors; WFH = work-from-home.

Notes 1. Other regressors not shown in the table include the road-based distance between the origin and destination (except model 3), number of working days in each week, whether the origin city’s lockdown was more or less stringent than that of destination city, numbers of cases in destination and origin cities in the past two weeks.

2. Standard errors are clustered at the destination-week level.

3. ***, **, * indicate significant coefficients at 1%, 5%, 10%, respectively.

Source: Authors’ calculations.

**Table 3B:** Effects of lockdown measures by destination city firm size composition (excluding within-barangay flows).

Variables	Models		
	1	2	3
Benchmark effects			
ECQ+MECQ	-0.536***	-0.418***	-0.429***
	(0.007)	(0.009)	(0.009)
GCQ	-0.342***	-0.202***	-0.217***
	(0.010)	(0.010)	(0.010)
MGCQ	-0.257***	-0.154***	-0.164***
	(0.006)	(0.007)	(0.007)
ECQ+MECQ dummy × Employment share of medium and large firms	-0.240***	-0.162***	-0.186***
	(0.020)	(0.016)	(0.017)
GCQ dummy × Employment share of medium and large firms	-0.160***	-0.107***	-0.118***
	(0.026)	(0.021)	(0.022)
MGCQ dummy × Employment share of medium and large firms	-0.168***	-0.096***	-0.106***
	(0.019)	(0.016)	(0.016)
Origin and destination dummies	Yes	Yes	No
AR(1)	No	Yes	Yes
Origin-destination dummy	No	No	Yes
Observations	931,233	902,924	902,924
Adjusted R-squared	0.264	0.322	0.385

AR(1) = autoregressive term, lag 1; ECQ = enhanced community quarantine; GCQ = general community quarantine; MECQ = modified enhanced community quarantine; MGCQ = modified general community quarantine; SE = standard errors; WFH = work-from-home.

Notes: 1. Other regressors not shown in the table include the road-based distance between the origin and destination (except model 3), number of working days in each week, whether the origin city’s lockdown was more or less stringent than that of destination city, numbers of cases in destination and origin cities in the past two weeks.

2. Standard errors are clustered at the destination-week level.

3. ***, **, * indicate significant coefficients at 1%, 5%, 10%, respectively.

Source: Authors’ calculations.

Second, Table 1A shows that a significant amount of OD pairs disappeared completely from the data when COVID-19 broke out and some of them re-emerged in later weeks when the pandemic eased with relaxed lockdown. We constructed a balanced panel of city pairs to take into account these ODs. The flows are set at 0 when they are not observed in the data for certain weeks. The estimates with this balanced panel are reported in Tables 4A and 4B.

**Table 4A:** Effects of lockdown measures by destination city industrial composition (balanced panel of city pairs).

Variables	Model 1		Model 2		Model 3	
	β	SE	β	SE	β	SE
Benchmark effects						
ECQ+MECQ	-0.454***	(0.023)	-0.321***	(0.020)	-0.425***	(0.019)
GCQ	-0.066**	(0.025)	-0.005	(0.020)	-0.078***	(0.020)
MGCQ	-0.082***	(0.022)	-0.009	(0.017)	-0.080***	(0.017)
ECQ+MECQ dummy × Employment share of industry category						
Primary	0.283***	(0.080)	0.244***	(0.068)	0.277***	(0.067)
Power, utilities, construction	-0.562***	(0.123)	-0.405***	(0.097)	-0.405***	(0.092)
Trade, transport	0.308***	(0.041)	0.212***	(0.033)	0.212***	(0.032)
Hospitality, recreation	-0.205**	(0.075)	-0.140*	(0.063)	-0.164**	(0.060)
WFH-friendly tertiary	-0.433***	(0.059)	-0.305***	(0.051)	-0.315***	(0.048)
Non-WFH-friendly tertiary	-0.332***	(0.076)	-0.202**	(0.064)	-0.209***	(0.061)
GCQ dummy × Employment share of industry category						
Primary	-0.089	(0.099)	-0.146	(0.082)	-0.099	(0.084)
Power, utilities, construction	-0.382**	(0.138)	-0.259*	(0.104)	-0.292**	(0.107)
Trade, transport	0.050	(0.051)	0.006	(0.040)	-0.012	(0.040)
Hospitality, recreation	-0.321***	(0.092)	-0.212**	(0.073)	-0.202**	(0.071)
WFH-friendly tertiary	-0.692***	(0.058)	-0.472***	(0.048)	-0.526***	(0.048)
Non-WFH-friendly tertiary	-0.230**	(0.088)	-0.051	(0.069)	-0.098	(0.070)
MGCQ dummy × Employment share of industry category						
Primary	0.140	(0.072)	0.074	(0.056)	0.111	(0.061)
Power, utilities, construction	-0.361**	(0.114)	-0.210*	(0.086)	-0.218*	(0.085)
Trade, transport	0.176***	(0.039)	0.077*	(0.030)	0.105***	(0.030)
Hospitality, recreation	-0.208**	(0.080)	-0.137*	(0.061)	-0.151*	(0.064)
WFH-friendly tertiary	-0.578***	(0.064)	-0.395***	(0.051)	-0.463***	(0.051)
Non-WFH-friendly tertiary	-0.183**	(0.064)	-0.114*	(0.050)	-0.100*	(0.051)
Origin and destination dummies	Yes		Yes		No	
AR(1)	No		Yes		Yes	
Origin-destination dummy	No		No		Yes	
Observations	1,149,138		1,108,514		1,108,514	
Adjusted R-squared	0.213		0.303		0.356	

AR(1) = autoregressive term, lag 1; ECQ = enhanced community quarantine; GCQ = general community quarantine; MECQ = modified enhanced community quarantine; MGCQ = modified general community quarantine; SE = standard errors; WFH = work-from-home.

Notes: 1. Other regressors not shown in the table include the road-based distance between the origin and destination (except model 3), number of working days in each week, whether the origin city’s lockdown was more or less stringent than that of destination city, numbers of cases in destination and origin cities in the past two weeks.

2. Standard errors are clustered at the destination-week level.

3. ***, **, * indicate significant coefficients at 1%, 5%, 10%, respectively.

Source: Authors’ calculations.

**Table 4B:** Effects of lockdown measures by destination city firm size composition (balanced panel of city pairs).

Variables	Models		
	1	2	3
Benchmark effects			
ECQ+MECQ	-0.410***	-0.287***	-0.399***
	(0.007)	(0.007)	(0.007)
GCQ	-0.171***	-0.079***	-0.167***
	(0.011)	(0.009)	(0.009)
MGCQ	-0.108***	-0.047***	-0.113***
	(0.007)	(0.006)	(0.006)
ECQ+MECQ dummy × Employment share of medium and large firms	-0.280***	-0.194***	-0.179***
	(0.022)	(0.018)	(0.018)
GCQ dummy × Employment share of medium and large firms	-0.180***	-0.102***	-0.107***
	(0.027)	(0.021)	(0.021)
MGCQ dummy × Employment share of medium and large firms	-0.175***	-0.090***	-0.099***
	(0.021)	(0.017)	(0.016)
Origin and destination dummies	Yes	Yes	No
AR(1)	No	Yes	Yes
Origin-destination dummy	No	No	Yes
Observations	1,149,138	1,108,514	1,108,514
Adjusted R-squared	0.211	0.303	0.355

AR(1) = autoregressive term, lag 1; ECQ = enhanced community quarantine; GCQ = general community quarantine; MECQ = modified enhanced community quarantine; MGCQ = modified general community quarantine.

Notes: 1. Other regressors not shown in the table include the road-based distance between the origin and destination (except model 3), number of working days in each week, whether the origin city’s lockdown was more or less stringent than that of destination city, numbers of cases in destination and origin cities in the past two weeks.

2. Standard errors are clustered at the destination-week level.

3. ***, **, * indicate significant coefficients at 1%, 5%, 10%, respectively.

Source: Authors’ calculations.

Third, certain degree of symmetry is expected in work-related mobility between morning inflows and afternoon outflows. Hence, we constructed an afternoon sample which contains all flows leaving the 300 cities between 4 p.m. and 10 p.m. to any city within 150 kilometers road-based distance. In regressions, we replace the destination (origin) city’s characteristics with those of the origin (destination) city. The estimation results are reported in Tables 5A and 5B.

**Table 5A:** Effects of lockdown measures by destination city industrial composition (afternoon flows leaving the top 300 cities).

Variables	Model 1		Model 2		Model 3	
	β	SE	β	SE	β	SE
Benchmark effects						
ECQ+MECQ	-0.622***	(0.008)	-0.536***	(0.010)	-0.555***	(0.011)
GCQ	-0.240***	(0.010)	-0.233***	(0.010)	-0.241***	(0.009)
MGCQ	-0.273***	(0.008)	-0.264***	(0.008)	-0.273***	(0.008)
ECQ+MECQ dummy × Employment share of industry category						
Primary	0.441***	(0.023)	0.394***	(0.022)	0.407***	(0.021)
Power, utilities, construction	-0.453***	(0.027)	-0.385***	(0.027)	-0.421***	(0.026)
Trade, transport	0.340***	(0.014)	0.302***	(0.013)	0.324***	(0.013)
Hospitality, recreation	-0.074***	(0.022)	-0.038	(0.020)	-0.040	(0.021)
WFH-friendly tertiary	-0.304***	(0.014)	-0.283***	(0.014)	-0.304***	(0.013)
Non-WFH-friendly tertiary	-0.314***	(0.018)	-0.303***	(0.017)	-0.335***	(0.016)
GCQ dummy × Employment share of industry category						
Primary	0.168***	(0.030)	0.106***	(0.030)	0.119***	(0.028)
Power, utilities, construction	-0.364***	(0.040)	-0.267***	(0.040)	-0.286***	(0.037)
Trade, transport	0.086***	(0.020)	0.101***	(0.020)	0.092***	(0.019)
Hospitality, recreation	-0.159***	(0.029)	-0.114***	(0.027)	-0.124***	(0.026)
WFH-friendly tertiary	-0.532***	(0.017)	-0.463***	(0.018)	-0.488***	(0.019)
Non-WFH-friendly tertiary	-0.310***	(0.031)	-0.325***	(0.032)	-0.335***	(0.030)
MGCQ dummy × Employment share of industry category						
Primary	0.177***	(0.026)	0.149***	(0.021)	0.154***	(0.020)
Power, utilities, construction	-0.373***	(0.029)	-0.269***	(0.029)	-0.297***	(0.028)
Trade, transport	0.199***	(0.014)	0.220***	(0.013)	0.223***	(0.013)
Hospitality, recreation	-0.101*	(0.042)	-0.090**	(0.027)	-0.101***	(0.028)
WFH-friendly tertiary	-0.475***	(0.019)	-0.425***	(0.018)	-0.437***	(0.018)
Non-WFH-friendly tertiary	-0.197***	(0.018)	-0.178***	(0.017)	-0.186***	(0.017)
Origin and destination dummies	Yes		Yes		No	
AR(1)	No		Yes		Yes	
Origin-destination dummy	No		No		Yes	
Observations	906,864		875,839		875,839	
Adjusted R-squared	0.263		0.307		0.369	

AR(1) = autoregressive term, lag 1; ECQ = enhanced community quarantine; GCQ = general community quarantine; MECQ = modified enhanced community quarantine; MGCQ = modified general community quarantine; SE = standard errors; WFH = work-from-home.

Notes: 1. Other regressors not shown in the table include the road-based distance between the origin and destination (except model 3), number of working days in each week, whether the origin city’s lockdown was more or less stringent than that of destination city, numbers of cases in destination and origin cities in the past two weeks.

2. Standard errors are clustered at the destination-week level.

3. ***, **, * indicate significant coefficients at 1%, 5%, 10%, respectively.

Source: Authors’ calculations.

**Table 5B:** Effects of lockdown measures by destination city firm size composition (afternoon flows leaving the top 300 cities).

Variables	Models		
	1	2	3
Benchmark effects			
ECQ+MECQ	-0.530***	-0.454***	-0.468***
	(0.004)	(0.007)	(0.008)
GCQ	-0.296***	-0.271***	-0.287***
	(0.005)	(0.006)	(0.006)
MGCQ	-0.273***	-0.242***	-0.253***
	(0.003)	(0.004)	(0.005)
ECQ+MECQ dummy × Employment share of medium and large firms	-0.247***	-0.222***	-0.242***
	(0.007)	(0.007)	(0.006)
GCQ dummy × Employment share of medium and large firms	-0.160***	-0.153***	-0.155***
	(0.009)	(0.010)	(0.009)
MGCQ dummy × Employment share of medium and large firms	-0.132***	-0.134***	-0.138***
	(0.007)	(0.007)	(0.006)
Origin and destination dummies	Yes	Yes	No
AR(1)	No	Yes	Yes
Origin-destination dummy	No	No	Yes
N	906,864	875,839	875,839
Adjusted R-squared	0.261	0.306	0.368

AR(1) = autoregressive term, lag 1; ECQ = enhanced community quarantine; GCQ = general community quarantine; MECQ = modified enhanced community quarantine; MGCQ = modified general community quarantine.

Notes: 1. Other regressors not shown in the table include the road-based distance between the origin and destination (except model 3), number of working days in each week, whether the origin city’s lockdown was more or less stringent than that of destination city, numbers of cases in destination and origin cities in the past two weeks.

2. Standard errors are clustered at the destination-week level.

3. ***, **, * indicate significant coefficients at 1%, 5%, 10%, respectively.

Source: Authors’ calculations.

Patterns of heterogeneous mobility reduction emerging from these exercises are qualitatively the same as those from the baseline case despite significant data variations. Lockdown effects on work-related mobility vary across cities with different industrial and firm size compositions. Cities with higher industrial concentration in utilities, construction, and tertiaries and with larger employers were more adversely affected. Moreover, mobility responses to lockdown measures of varying stringency also depended on the employment characteristics of a city. When less stringent lockdown was in place, more mobility recovery occurred in cities heavy in non-WFH-friendly and power, utilities and construction sectors, and less so in cities with more WFH-friendly sectors.

Meanwhile, there are a couple of noteworthy differences between the results of the sensitivity and baseline analyses. The relative effects for share of hospitality and recreation employment and share of WFH-friendly employment increase significantly in magnitude when the balanced panel is used to include disappeared city pairs. This suggests that industries in these two categories (e.g., restaurants and call centers) served as the major economic connectivity between some cities, which could have been cut down during lockdowns. Besides, we find larger benchmark effects of mobility reduction under all three lockdown policies with PM outflows than AM inflows. This may be because the working hours were shortened, and some commuters left their workplace early.

## Discussion and conclusion

While NPIs have proved to be effective in reducing mobility to control COVID-19 spread in advanced economies, there is little evidence on whether and how similar measures have worked in lower-income countries. An equally important question is how they have impacted the economy. This study sheds light on these questions through studying the lockdown policies within 6 months since the breakout of COVID-19 in the Philippines.

Consistent with findings from advanced economies, community quarantines were an effective policy for reducing urban mobility in the Philippines during crises, and mobility was highly responsive to different degrees of lockdown stringency. By design and in practice, lockdown measures were less effective in limiting mobility for manufacturing-based cities. This could be economically rational, as keeping the industrial sector open lessens economic costs without worsening health outcomes because industrial activities are less contact intensive [23].

As main target of lockdown policies, the tertiary sector was more severely affected (aside from trade and transport industries). However, given the diversity of jobs and businesses within tertiary sector, we see distinct policy impacts on cities which have a higher share of workforce in industries amenable versus not amenable to working from home. Low-skilled service workers could still suffer from the aftereffects of lockdowns when returning to the workplace, as they were dependent on demand from workers who have shifted to home office and stayed there even under less stringent lockdown [24]. Policymakers may want to take this into account in designing assistance programs or deciding allocation of resources within their jurisdictions. More livelihood support may be needed to cities and municipalities that have a larger population share working in non-WFH-friendly tertiary industries.

Cities relying on tourism wherein hospitality and recreation workers cluster also need special policy attention. Although our results suggest these cities were subject to moderate additional mobility reduction due to lockdown, the actual loss of business could be substantial as people are sensitive to pandemic threat more than to policy restrictions, and our data do not capture long-distance (including international) travelers, which have important economic implications for many tourism-based cities. Programs that help these workers to transfer to other sectors that reopened or newly developed may be warranted to mitigate the adverse impacts of COVID-19 and lockdown policies.

The finding that lockdown measures were less effective in cities dominated by micro and small firms is also informative. Employees of these firms have lower income and limited access to health and social security services in general. They are thus exposed to greater health and financial risks when continuing to work under lockdown. Supplementary NPIs may be needed to protect them from infection and financial support to ensure their access to necessary health services.

Looking ahead, as health and climate-related disasters increase in prevalence, public interventions including social safety nets need to be designed more proactively. An understanding of the implications of emergency measures for cities of differing economic profiles can contribute to efficient utilization of public resources.

Finally, our study demonstrates the usefulness of big data for understanding policy impacts and supporting informed policymaking. Its granularity in space and time complements traditional data, such as those based on large-scale household or firm surveys. On the other hand, big data, including the cellphone-based flow data used here, often contain few variables and require merging with other data sources for deeper policy analysis. In particular, they typically lack direct measures of economic outcomes of individuals or households, which limit researchers’ ability to assess cost-effectiveness of policies or conduct social welfare analysis. Linking the two types of data will allow for more meaningful and relevant analyses in the future.

## Supporting information

S1 Fig Hourly profiles of total flows by month. (DOCX)

S1 Data (ZIP)

## References

[1] Hale T, Angrist N, Goldszmidt R, Kira B, Petherick A, Phillips T, et al. A global panel database of pandemic policies (Oxford COVID-19 Government Response Tracker). Nature Human Behaviour. 2021. 10.1038/s41562-021-01079-8 33686204

[2] Glaeser EL, Gorback C, Redding SJ. JUE insight: how much does COVID-19 increase with mobility? evidence from New York and four other US cities. Journal of Urban Economics. 2020;103292. 10.1016/j.jue.2020.103292 PMC7577256 33106711

[3] Hadjidemetriou GM, Sasidharan M, Kouyialis G, Parlikad AK. The impact of government measures and human mobility trend on COVID-19 related deaths in the UK. Transportation Research Interdisciplinary Perspectives. 2020;6:100167. doi: 10.1016/j.trip.2020.100167 34173458 PMC7334915

[4] Beland L-P, Brodeur A, Wright T. COVID-19, stay-at-home orders and employment: evidence from CPS data. IZA Discussion Paper. 2020.

[5] Chen S, Igan DO, Pierri N, Presbitero AF, Soledad M, Peria M. Tracking the economic impact of COVID-19 and mitigation policies in Europe and the United States. IMF Working Papers. 2020;125.

[6] Sampi Bravo JRE, Jooste C. Nowcasting economic activity in times of COVID-19: an approximation from the Google Community Mobility Report. World Bank Policy Research Working Paper. 2020;9247.

[7] Lee M, Zhao J, Sun Q, Pan Y, Zhou W, Xiong C, et al. Human mobility trends during the early stage of the COVID-19 pandemic in the United States. PLoS One. 2020;15(11):e0241468. doi: 10.1371/journal.pone.0241468 33166301 PMC7652287

[8] Hu S, Xiong C, Yang M, Younes H, Luo W, Zhang L. A big-data driven approach to analyzing and modeling human mobility trend under non-pharmaceutical interventions during COVID-19 pandemic. Transportation Research Part C: Emerging Technologies. 2021;124:102955. doi: 10.1016/j.trc.2020.102955 33456212 PMC7796660

[9] Pullano G, Valdano E, Scarpa N, Rubrichi S, Colizza V. Population mobility reductions during COVID-19 epidemic in France under lockdown. MedRxiv. 2020. 10.1016/S2589-7500(20)30243-0 PMC7598368 33163951

[10] Aloi A, Alonso B, Benavente J, Cordera R, Echániz E, González F, et al. Effects of the COVID-19 lockdown on urban mobility: empirical evidence from the city of Santander (Spain). Sustainability. 2020;12(9):3870.

[11] Heiler G, Reisch T, Hurt J, Forghani M, Omani A, Hanbury A, et al. Country-wide mobility changes observed using mobile phone data during COVID-19 pandemic. IEEE International Conference on Big Data. 2020;pp.3123-3132.

[12] Dahlberg M, Edin P-A, Grönqvist E, Lyhagen J, Östh J, Siretskiy A, et al. Effects of the COVID-19 pandemic on population mobility under mild policies: causal evidence from Sweden. arXiv preprint arXiv. 2020;2004.09087.

[13] Caselli M, Fracasso A, Scicchitano S. From the lockdown to the new normal: an analysis of the limitations to individual mobility in Italy following the Covid-19 crisis. GLO Discussion Paper. 2020;683.

[14] Hara Y, Yamaguchi H. Japanese travel behavior trends and change under COVID-19 state-of-emergency declaration: nationwide observation by mobile phone location data. Transportation Research Interdisciplinary Perspectives. 2021;9:100288. doi: 10.1016/j.trip.2020.100288 34173482 PMC7833687

[15] David AC, Pienknagura S. On the effectiveness of containment measures in controlling the COVID-19 pandemic: the role of labour market characteristics and governance. Applied Economics Letters. 2020;1–7.

[16] Askitas N, Tatsiramos K, Verheyden B. Lockdown strategies, mobility patterns and covid-19. Working paper. 2020.

[17] Camehl A, Rieth M. Disentangling Covid-19, economic mobility, and containment policy shocks. Working paper. 2021.

[18] Wang S, Tong Y, Fan Y, Liu H, Wu J, Wang Z, et al. Observing the silent world under COVID-19 with a comprehensive impact analysis based on human mobility. Scientific Reports. 2021;11(1):1–12. doi: 10.1038/s41598-021-94060-4 34282180 PMC8289815

[19] Goldstein P, Levy Yeyati E, Sartorio L. Lockdown fatigue: the diminishing effects of quarantines on the spread of COVID-19. Working paper. 2021.

[20] Eckert F, Ganapati S, Walsh C. Skilled Scalable Services: the new urban bias in economic growth. 2020; SSRN 3439118.

[21] Dingel J, Brent N. How many jobs can be done at home?. Journal of Public Economics. 2020;189:104235. doi: 10.1016/j.jpubeco.2020.104235 32834177 PMC7346841

[22] Inter-Agency Task Force for the Management of Emerging Infectious Diseases (IATF) [Internet]. Official Gazette; 2020. Omnibus guidelines on the implementation of community quarantine in the Philippines with amendments as of October 8, 2020; [cited 2020 October 10]. https://www.officialgazette.gov.ph/downloads/2020/10oct/20201008-IATF-Omnibus-Guidelines-RRD.pdf.

[23] Furceri D, Kothari S, Zhang L. The effects of COVID-19 containment measures on the Asia-Pacific region. Pacific Economic Review. 2021.

[24] Althoff L, Eckert F, Ganapati S, Walsh C. The geography of remote work. National Bureau of Economic Research. 2021;w29181.

