# Supplementary figures and images for: COVID-19 lockdown policy and heterogeneous responses of urban mobility: Evidence from the Philippines
Source: PLoS One. 2022 Jun 30;17(6):e0270555. doi: 10.1371/journal.pone.0270555 (PMC9246172; doi:10.1371/journal.pone.0270555)

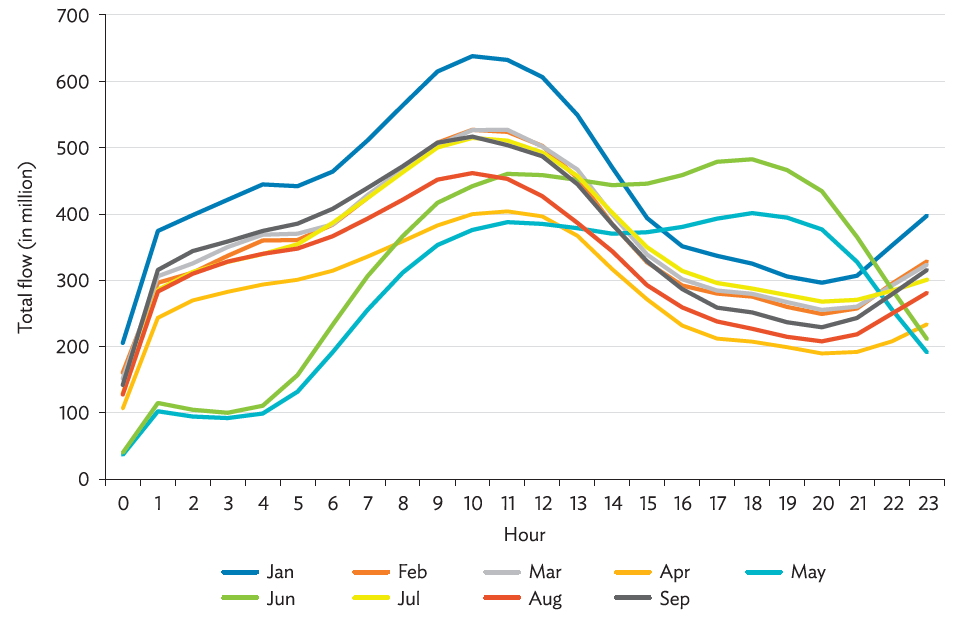


Source: Authors’ calculations.

Supplement: S1 Fig — (DOCX) [file pone.0270555.s001.docx]
