# Supplementary material for: COVID-19 lockdown policy and heterogeneous responses of urban mobility: Evidence from the Philippines
Source: PLoS One. 2022 Jun 30;17(6):e0270555. doi: 10.1371/journal.pone.0270555 (PMC9246172; doi:10.1371/journal.pone.0270555)
Supplement: S1 Data — (ZIP) [file pone.0270555.s002.zip › Data availability.docx]

“COVID-19 Lockdown Policy and Heterogeneous Responses of Urban Mobility: Evidence from the Philippines” by Yi Jiang, Jade R. Laranjo, Milan Thomas

Data Availability

- Two Stata datasets are provided here: “Urban mobility in PHI - AM sample.dta” and “Urban mobility in PHI - PM sample.dta”. They contain variables on cellphone-based weekly flows, quarantine policies and city/municipality-level characteristics including infection counts. Not included is the city/municipality employment by sector and firm size, which is constructed using List of Establishment (LE) 2018 data. The authors are not authorized to publicize the LE data, but it could be accessed by contacting Philippine Statistics Authority (https://psa.gov.ph/directory).
- Combined with the LE data, “Urban mobility in PHI - AM sample.dta” can be used to replicate all results in Tables 1A, 1B, 2A, 2B, 3A, 3B, 4A and 4B of the subject paper; “Urban mobility in PHI - PM sample.dta” can be used to replicate all results in Tables 5A and 5B.
- The datasets should be used solely for replicating the results of the subject study. Other use of the datasets is not permitted without obtaining authors’ agreement in advance.
